# A Formative Evaluation of Parental Perceptions Related to Acceptability, Appropriateness, Feasibility, and Reported Use of an e-Learning Resource Targeting Diet in the First 1000 Days: Survey Study

**DOI:** 10.2196/84277

**Published:** 2026-04-28

**Authors:** Natalie Garzon Osorio, Frøydis Nordgård Vik, Elisabet Rudjord Hillesund, Sissel Heidi Helland, Penelope Love, Marianne Hope Abel, Harry Rutter, Andrew Keith Wills, Christine Helle, Tormod Bjørkkjær, Nina Cecilie Øverby, Anine Christine Medin

**Affiliations:** 1 Department of Nutrition and Public Health, Faculty of Health and Sport Sciences, University of Agder, Universitetsveien 25, Kristiansand, 4630, Norway, +4799570261; 2 Institute for Physical Activity and Nutrition, School of Exercise and Nutrition Sciences, Deakin University, Geelong, VIC, Australia; 3 Centre for Evaluation of Public Health Measures, Norwegian Institute of Public Health, Oslo, Norway; 4 Centre for 21st Century Public Health, University of Bath, Bath, United Kingdom

**Keywords:** digital health, e-learning resource, digital intervention, diet intervention, Acceptability of Intervention Measure, AIM, Intervention Appropriateness Measure, IAM, Feasibility of Intervention Measure, FIM, first 1000 days

## Abstract

**Background:**

In October 2022, the Nutrition Now (NN) e-learning resource was implemented within Maternal and Child Healthcare centers and Early Childhood Education and Care centers of a southern Norwegian municipality. The e-learning resource targets expectant parents, parents of children aged 0‐2 years, and Early Childhood Education and Care staff, aiming to promote healthy dietary behaviors during the first 1000 days of life.

**Objective:**

This study aimed to explore parental perceptions related to the acceptability, appropriateness, feasibility, and reported use of the NN e-learning resource among parents.

**Methods:**

From October 2022 to May 2023, expecting parents and parents of children aged 0‐2 years were recruited from 2 Norwegian municipalities, one intervention group receiving access to the NN e-learning resource, and one control. Participants in the intervention group received a web-based follow-up questionnaire 7 months after gaining access to the NN e-learning resource. Data were analyzed using descriptive statistics.

**Results:**

Of the 179 participants in the NN study intervention group, 48 completed the web-based follow-up questionnaire administered 7 months after enrollment. Parents rated the e-learning resource positively on items assessing whether they liked and appreciated the resource, perceived it as an appropriate source of information, and found it doable and easy to use. Most respondents reported visiting the resource (38/48, 79%), although only 21% (10/48) reported frequent visits. Less than half of the participants answering the web-based follow-up questionnaire reported having watched the theme films (20/48, 42%), the recipe films (17/48, 35%), or making food using recipes provided in the e-learning resource (20/48, 42%).

**Conclusions:**

Parents rated the NN e-learning resource positively but reported limited use. These findings point to the need for strategies that enhance engagement with self-guided digital interventions among expectant parents and parents of young children. Future efforts should focus on identifying how to maximize potential adoption of the e-learning resource and evaluate its impact to promote healthy dietary behaviors during the first 1000 days of life.

## Introduction

A balanced diet in early life is important for children’s current and future health [1-3], with parents serving as the primary influences on their dietary behaviors. Digital health resources may have the potential to provide parents with accessible tools to support healthy diets within families [4,5]. Systematic reviews evaluating parent-focused digital and mobile health (mHealth) interventions suggest that some interventions may improve selected dietary behaviors, such as fruit and vegetable intake or reduced sugar-sweetened beverage consumption, while others show no significant effect [4,6]. Furthermore, few of these interventions target pregnant women or children younger than 2 years of age. The period from conception to a child’s second birthday, commonly referred to as “the first 1000 days,” represents a critical window during which the foundations for healthy dietary habits are established [7,8]. Developing and evaluating digital interventions targeting this early-life period could therefore be important for both research and public health.

To promote healthy dietary behaviors in expecting parents and families with infants and toddlers, the Nutrition Now (NN) e-learning resource was developed and made available through Maternal and Child Healthcare (MCH) and Early Childhood Education and Care (ECEC) centers in a southern Norwegian municipality [9]. The e-learning resource combines content from 4 dietary interventions with existing evidence of effectiveness [10-14] into a single comprehensive web-based platform, targeting different early-life transitions from pregnancy to the age of 2 years. In 2 of the interventions underpinning the NN e-learning resource, parents were involved in the development of intervention content by providing feedback on intervention materials and participating in surveys and qualitative interviews about their preference for content and experiences with the interventions [15,16]. Insights from these evaluations informed the tailoring of the NN e-learning resource. The content of the NN e-learning resource was primarily designed for parents and ECEC staff but was also accessible for MCH staff. The part of the e-learning resource targeting parents was organized into four stages, including (1) pregnancy, (2) newborn, (3) infants aged 5‐12 months, and (4) toddlers aged 1‐2 years. The e-learning resource provided content tailored to these stages, presented through (1) short thematic films and texts covering topics such as diet during pregnancy, the introduction of solid foods, and the development of healthy eating habits; (2) cooking videos; and (3) age-appropriate recipes. Screenshots from the e-learning resource are presented in Multimedia Appendix 1. A detailed description of the NN e-learning resource and its components is provided in the study protocol [9].

Although digital health interventions have shown potential to improve dietary behaviors, much of the existing evidence is derived from controlled research trials, and there remains a limited understanding of how such interventions perform under real-world settings. Evaluating implementation outcomes is therefore important for identifying facilitators and barriers for effective implementation, and subsequently, intervention effectiveness [17]. In the context of digital health interventions, assessing outcomes such as acceptability, appropriateness, and feasibility may provide important insights into how interventions are received and whether they are likely to be adopted and sustained in practice [18]. In a systematic review by Bonvicini et al [6], assessing the effectiveness of mHealth interventions targeting parents to prevent and treat childhood obesity, the authors report that all studies (n=20) evaluated usability, acceptability, and feasibility of their interventions. However, there remain inconsistencies in how these outcomes are defined and measured across studies within digital health. Using standardized definitions and evaluation criteria to assess these outcomes may help in faster translation of how digital health interventions succeed or fail in their effectiveness to change behavior [18,19]. Implementation science offers several frameworks for the evaluation of implementation outcomes, including the framework by Proctor et al [17], proposing 8 key outcomes: acceptability, appropriateness, adoption, feasibility, fidelity, penetration, cost, and sustainability [20]. Acceptability, appropriateness, and feasibility are commonly explored during the earlier stages of implementation [21,22] and can help tailor interventions and implementation strategies to user needs and preferences, potentially enhancing intervention adoption and impact [17,23]. According to the implementation outcomes framework of Proctor et al [17], acceptability is the perception among users that an intervention is agreeable, palatable, or satisfactory. Appropriateness is defined as the perceived fit, relevance, or compatibility of the intervention for a given person and/or the perceived fit of the intervention to address a particular issue or problem [17]. Feasibility is the extent to which an intervention can be successfully used or carried out within a given setting and reflects actual fit or suitability for everyday use [17]. Although these outcomes are conceptually similar, they capture different dimensions of early implementation [17]. An intervention may be perceived as appropriate but not feasible, or acceptable but not appropriate, underscoring the importance of assessing these outcomes separately [23].

Furthermore, it is important to examine intervention use to identify features that are most or least engaging. Intervention use is often described in the context of engagement, which is conceptualized in a variety of ways within digital intervention research [24]. Perski et al [25] have proposed a 2-part definition of engagement, where the two key components are (1) extent of use and (2) subjective experience. According to Perski, the extent of use is determined by the length of intervention contact, time exposed to the intervention, frequency of contact, and variety of content used. Together, acceptability, appropriateness, feasibility, and intervention use capture complementary aspects important to understand early implementation processes. Acceptability, appropriateness, and feasibility reflect users’ perceptions about an intervention and may influence intentions to adopt and extent of use [23]. On the other hand, extent of use and experience with an intervention may shape perceptions of fit or relevance [26]. Evaluating both perceived implementation outcomes and aspects of actual use thus offers important insight into factors potentially influencing intervention effectiveness.

Finally, when evaluating digital health interventions, it is relevant to explore the characteristics of the user to determine if the intended user groups are being reached [27].

The aim of this study was to conduct a formative evaluation exploring parental perceptions related to acceptability, appropriateness, feasibility, and reported use of the NN e-learning resource among expectant parents and parents of children aged 0‐2 years.

## Methods

### Study Setting, Participant Recruitment, and Data Collection

The NN project is a hybrid type 1 implementation-effectiveness trial, using a quasi-experimental, nonrandomized design. One Norwegian municipality received access to the NN e-learning resource, while a control municipality was not given access and received usual care [9]. The NN project comprises 2 phases, with the first phase encompassing implementation within a municipality setting before scale-up to county settings (phase 2) [9]. This study reports descriptive findings from a formative evaluation conducted during the first phase of the NN project, focusing on parents in the intervention municipality who were invited to use the NN e-learning resource. The findings were intended to inform refinement of the resource and its implementation strategies before upscaling in phase 2. All expectant parents and parents of children aged 0‐2 years were eligible to participate. This study adhered to the CHERRIES (Checklist for Reporting Results of Internet E-Surveys) [28], and the completed checklist is available in Checklist 1.

Recruitment occurred between October 2022 and May 2023 through MCH centers, ECEC centers, municipal websites, and social media. Eligible participants completed a web-based baseline questionnaire providing written informed consent and data on sociodemographic characteristics. Once enrolled, parents in the intervention municipality gained free access to the full e-learning resource. The e-learning resource was designed for the general population of expectant parents and parents of children aged 0‐2 years, and its universal design aimed to make it accessible to families across socioeconomic positions and ethnic backgrounds.

The parent modules within the e-learning resource were self-guided and designed to be independent of human support [29,30]. Content was available in Norwegian, English, and Arabic. To encourage engagement, participants received email newsletters linking directly to the NN e-learning resource, with pregnancy-specific content for those enrolled during pregnancy and age-appropriate content for those with children. In total, 4 newsletters were sent during pregnancy, 1 at birth, 8 sent monthly from 4 to 12 months, and 12 sent biweekly from 12 to 24 months. These newsletters were automatically distributed based on the due date or the child’s birthdate provided at baseline. Although newsletters provided links to age-relevant content based on due date or child’s age, parents were free to navigate and use any part of the resource.

Seven months after enrollment, participants were invited by email to complete a web-based follow-up questionnaire on implementation-related outcomes and e-learning resource use. Participants who did not respond to the initial invitation received 3 additional email reminders. As the e-learning resource was self-guided and the fact that parents were unrestricted in the content they accessed, responses to the follow-up questionnaire reflect parents’ perceptions based on the components they chose to engage with.

All data were collected using digital questionnaires created in the survey tool Nettskjema (University of Oslo), integrated with Services for Sensitive Data (TSD, University of Oslo), ensuring secure collection, storage, and analysis of sensitive research data. Data were pseudonymized using numerical identifiers, and access to identifiable information was restricted to members of the research team.

### Variables and Assessment

The survey questions used in this study are available in Tables S1 and S2 in Multimedia Appendix 2. Information on sociodemographic characteristics was obtained through 9 questions in the web-based baseline questionnaire, which included queries about parental gender, country of birth, employment status, urban or rural residence, civil status, household size, household income, and educational level.

For this study, items informed by the Acceptability of Intervention Measure (AIM), Intervention Appropriateness Measure (IAM), and Feasibility of Intervention Measure (FIM), developed by Weiner et al [23], were used to describe parental perceptions of the e-learning resource. The original measures contained 4 items, each rated on a 5-point Likert scale from 1=completely disagree to 5=completely agree, with higher scores indicating greater acceptability, appropriateness, or feasibility. These measures were selected because they provide brief instruments specifically developed to assess implementation outcomes aligned with the framework proposed by Proctor et al [17,23]. Although several other measurement instruments exist to assess implementation outcomes, the AIM, IAM, and FIM are among the measures with the fewest items for assessing these early-implementation outcomes [31]. Additionally, they are not restricted to a particular intervention type or context [23]. For these reasons, they were considered the most appropriate starting point for developing a tailored questionnaire for parents having access to the NN e-learning resource.

In this study, unpublished Norwegian translations of the AIM, IAM, and FIM measures were used. During questionnaire development, the research team found several items to be difficult to understand in Norwegian, both in terms of wording and conceptual clarity. It was considered likely that our target group (expectant parents and parents of children aged 0‐2 years) could encounter similar challenges. To reduce redundancy and improve clarity, multiple items were excluded after thorough discussion within the team. The final version used for this study retained only the third and fourth items from the original AIM and FIM, and the first item of the IAM, and was rephrased for clarity. While these changes may have affected the measures’ validity, they were necessary to improve clarity and reduce respondent burden. Given the reduction and rephrasing of items, the adapted questions used in this study no longer reflect the original multi-item measures. For this reason, responses are analyzed and presented at the item level and interpreted as parents’ ratings of whether they liked the e-learning resource, appreciated it as a new resource, believed it was appropriate as a source of information regarding meals, found it doable, and found it easy to use.

In line with the definition of engagement of Perski et al [25], with distinctions between subjective experience and extent of use, this study focuses on the latter, with emphasis on frequency (ie, how often contact is made with the intervention over time) and depth (ie, variety of content used). Although objective platform analytics (eg, number of logins and time spent on specific pages) were available for the NN e-learning resource, these data could not be linked to individual participants. Use was therefore assessed through self-report measures. Specifically, to evaluate use of the e-learning resource, parents were asked whether they had visited it, with response options including “Yes, quite a lot,” “Yes, a bit,” or “No.” These options were designed to capture participants’ self-perceived use without imposing specific frequency thresholds, aiming to reflect broad individual use patterns. Participants answering “Yes, a bit” or “No” were asked a follow-up question about why they had not visited the NN e-learning resource or used it sparingly. Response options included “Didn’t have time,” “Had technical problems,” “Lost interest,” and “Other,” with the latter option allowing participants to provide a written explanation. Additionally, parents were asked whether they had watched the thematic films or recipe films and whether they had used any of the recipes provided in the e-learning resource, with response options including “Yes,” “No,” and “Don’t know.”

### Data Analysis

Descriptive statistics (frequencies with percentages, means with SDs, and medians with IQRs) were used to summarize data related to demographic characteristics; responses to the individual acceptability-, appropriateness-, and feasibility-related items; and reported use of the e-learning resource. Internal consistency of the adapted 2-item AIM and FIM measures was assessed using the Cronbach α coefficient. Construct validity of the IAM item was examined using Spearman ρ correlation with the mean scores of the adapted AIM and FIM measures. The results are presented in Table S1 in Multimedia Appendix 3.

Since many participants were lost to follow-up, differences in demographic characteristics were assessed between participants answering the follow-up administered 7 months after enrollment and participants lost to follow-up. This was done using the chi-square test and Fisher exact test due to indications of nonnormality.

All statistical procedures were performed with SPSS (version 29; IBM Corp).

### Ethical Considerations

This study is part of the NN project, approved by the regional committees for Medical Research Ethics South East Norway (reference: 32248), the University of Agder’s Faculty Ethical Committee, and the Norwegian Data Protection Service (reference: 847590). All participants who wished to take part in the NN study were directed to the study’s registration website, where participants were informed about the study’s aim and their participation rights, including their right to withdraw from the study at any time. Participation was voluntary, and electronic informed consent was obtained when participants chose to sign up for the study. Participants could withdraw from the study at any time. To protect participant privacy, all data were pseudonymized using unique numerical identifiers, and directly identifiable information was accessible only to authorized members of the research team. No personally identifiable information is presented in the paper. Participants in the intervention municipality did not receive compensation for participation in the study or for completing the questionnaire administered 7 months after enrollment.

## Results

In total, 179 participants enrolled in the NN study, of whom 48 (27%) responded to the web-based follow-up questionnaire administered 7 months after enrollment, and 131 (73%) were lost to follow-up. An overview of their characteristics is presented in Table 1. Most participants were Norwegian-born women working or on leave of absence and residing in urban areas. The majority were cohabiting or married, living in 3-person households, with annual household incomes between US $91,726 and US $123.478. A difference in educational attainment was observed between those who completed the web-based follow-up questionnaire and those who did not respond. A higher proportion of follow-up respondents reported that either they or their partner had higher education compared to nonresponders (44/48, 92% vs 93/131, 71%), suggesting differential attrition by educational level.

**Table 1.** Baseline characteristics for participants in the Nutrition Now study intervention group, including those answering a web-based follow-up questionnaire administered 7 months after enrollment and those lost to follow-up.

Baseline characteristics	Total (N=179)^a^, n (%)	Participants at follow-up (n=48)^b^, n (%)	Participants lost to follow-up (n=131)^c^, n (%)	*P* value
Gender				.99^d^
Women	157 (88)	42 (88)	115 (88)	
Men	15 (8)	4 (8)	11 (8)	
Missing	7 (4)	2 (4)	5 (4)	
Country of birth				.09^d^
Norway	141 (79)	42 (88)	99 (76)	
Other	31 (17)	4 (8)	27 (21)	
Missing	7 (4)	2 (4)	5 (4)	
Employment status				.57^e^
Working	76 (43)	18 (38)	58 (44)	
Student	10 (6)	2 (4)	8 (6)	
Leave of absence	62 (35)	17 (35)	45 (34)	
Other	24 (13)	9 (19)	15 (12)	
Missing	7 (4)	2 (4)	5 (4)	
Residential area				.99^d^
Urban living	139 (78)	37 (77)	102 (78)	
Rural living	33 (18)	9 (19)	24 (18)	
Missing	7 (4)	2 (4)	5 (4)	
Civil status				.79^e^
In relationship	2 (1)	—^f^	2 (2)	
Cohabitant	88 (49)	24 (50)	64 (49)	
Married	78 (44)	22 (46)	56 (43)	
Other	4 (2)	—	4 (3)	
Missing	7 (4)	2 (4)	5 (4)	
Number of people in household				.97^e^
1	2 (1)	—	2 (2)	
2	25 (14)	6 (13)	19 (15)	
3	81 (45)	21 (44)	60 (46)	
4	44 (25)	13 (27)	31 (24)	
≥5	20 (11)	6 (13)	14 (11)	
Missing	7 (4)	2 (4)	5 (4)	
Household income (EUR)^g^				.17^e^
<€52,000	24 (13)	3 (6)	21 (16)	
€52,000‐€78,000	40 (22)	9 (19)	31 (24)	
€78,000‐€105,000	60 (34)	22 (46)	38 (29)	
>€105,000	45 (25)	11 (23)	34 (26)	
Do not want to answer	3 (2)	1 (2)	2 (2)	
Missing	7 (4)	2 (4)	5 (4)	
Educational level^h^				.007^e^
University or college over 4 years	83 (46)	25 (52)	58 (44)	
University or college up to 4 years	54 (30)	19 (40)	35 (27)	
Upper secondary school	33 (18)	2 (4)	31 (24)	
Primary and lower secondary school	2 (1)	—	2 (2)	
Missing	7 (4)	2 (4)	5 (4)	

a In total, 179 participants from the intervention municipality enrolled in the Nutrition Now study and received access to the Nutrition Now e-learning resource. All intervention participants were eligible to receive the web-based follow-up questionnaire administered 7 months after enrollment. Data on sociodemographic characteristics is lacking for 7 (4%) participants at baseline.

b Participants at follow-up are defined as expectant parents and parents of children aged 0‐2 years enrolling in the study and answering the follow-up questionnaire administered 7 months after enrollment. Within the participant in the follow-up group, there are missing values on all variables for 2 (4%) participants at baseline.

c Participants lost to follow-up are defined as expectant parents and parents of children aged 0‐2 years enrolling in the study and not answering the follow-up questionnaire administered 7 months after enrollment. Within the participant lost to follow-up group, there are missing values on all variables for 5 (4%) participants at baseline.

d Chi-square test with Yates continuity correction.

e Fisher exact test.

f Not applicable.

g Household income is calculated from Norwegian Kroners (NOK) to EUR using an exchange rate of 11.46 as of July 2, 2024. Income figures have been rounded up to the nearest thousand. A currency exchange rate of €1=US $1.18 is applicable.

h Educational level represents the highest attained educational level of at least 1 parent or caregiver.

**Figure 1.**
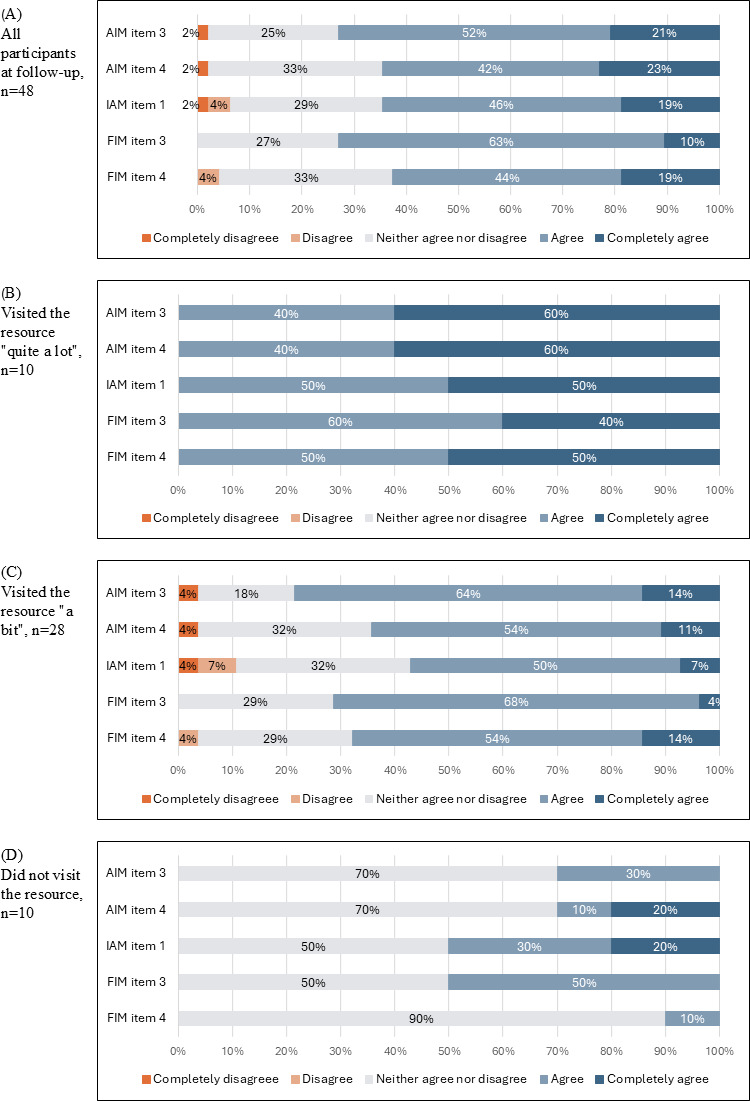
The proportion of responses to individual items informed by the AIM, IAM, and FIM among participants at follow-up. AIM item 3 refers to the statement “I like the Nutrition Now e-learning resource.” AIM item 4 refers to the statement “I appreciate Nutrition Now as a new resource.” IAM item 1 refers to the statement “Nutrition Now seems appropriate as a source of information regarding meals for my family.” FIM item 3 refers to the statement “The Nutrition Now resource seems doable.” FIM item 4 refers to the statement “The Nutrition Now resource seems easy to use.” (A) Proportion of responses from all participants. (B) Proportion of responses among participants visiting the e-learning resource “quite a lot.” (C) Proportion of responses among participants visiting the e-learning resource “a bit.” (D) Proportion of responses among participants not visiting the e-learning resource. AIM: Acceptability of Intervention Measure; FIM: Feasibility of Intervention Measure; IAM: Intervention Appropriateness Measure.

The median rating for the individual items assessing whether participants liked and appreciated the resource, perceived it as appropriate, and found it doable and easy to use was 4 (IQR 3-4; Table S2 in Multimedia Appendix 3), indicating that respondents generally agreed with the statements about the e-learning resource. The proportion of responses to all items is presented in Figure 1.

Table 2 presents participants’ use of the NN e-learning resource after 7 months of access, including whether they reported visiting the resource, reasons for not visiting, and whether they watched theme films, recipe films or used the provided recipes.

**Table 2.** Use of the Nutrition Now (NN) e-learning resource among participants answering a web-based follow-up questionnaire administered 7 months after enrollment.

Variables	Values (n=48), n (%)
Visited the resource	
Yes, quite a lot	10 (21)
Yes, a bit	28 (58)
No	10 (21)
Reasons for not visiting the resource^a^	
Did not have time	14 (29)
Had technical problems	2 (4)
Lost interest	6 (13)
Other^b^	16 (33)
Watched theme films	20 (42)
Watched recipe films	17 (35)
Made food using NN recipes	20 (42)

a Follow-up question provided to those answering “yes, a bit” or “no” to whether they had visited the resource.

b Participants answering “other” could provide a written response if they pleased. In total, 9 participants provided a response. Cited reasons among participants included forgetting about the resource, finding information elsewhere, not taking the time to visit, and finding it cumbersome to navigate.

## Discussion

### Principal Findings

The results show that participants receiving access to the NN e-learning resource and responding to the follow-up survey reported positive ratings regarding liking the e-learning resource, appreciating it as a new resource, finding it appropriate as a source of information regarding meals for their family, and finding it doable and easy to use. However, despite the positive ratings, the extent of self-reported use was limited. The majority of respondents (38/48, 79%) reported visiting the resource, but only 21% (10/48) reported frequent visits. Less than half reported watching the films (theme films: 20/48, 42% and recipe films: 17/48, 35%) or using the recipes provided in the e-learning resource (20/48, 42%). Given the low follow-up rate, small sample, and higher educational attainment among respondents, the findings should be interpreted with caution.

Our findings are consistent with previous studies on parent-focused digital health interventions to promote healthy diets. For example, Vázquez-Paz et al [32] reported that parents found the PersuHabit app, designed to promote healthy eating in children, to be likeable, useful, and easy to use. Similarly, Bonvicini et al [6] found that mHealth interventions targeting childhood obesity were generally well-received among parents across multiple studies. However, although digital interventions targeting parents and their children are positively perceived, maintaining engagement may be more difficult to achieve. In this study, although most parents reported visiting the NN e-learning resource, use of its components was limited. Only 21% (10/48) of respondents reported visiting the e-learning resource “a lot,” and less than half engaged with the theme films, recipe films, or used the provided recipes. These results contrast with those of the Early Food for Future Health study by Helle et al [11], where 85% of participants watched most of the videos, and 66% engaged with the cooking videos. Instead, our findings are more in line with the Food4Toddlers study by Røed et al [33], where participants completed only 8 of 22 lessons, with less than half trying 1‐5 recipes, and one-third using none.

Participants cited time constraints, loss of interest, and forgetfulness as reasons for limited use of the e-learning resource. These reasons may reflect broader contextual factors shaping parents’ capacity to engage with digital interventions during early parenthood. This life stage may be characterized by new routines and shifting priorities, which may reduce parents’ willingness or ability to explore an e-learning resource. In a previous qualitative evaluation of staff experiences with implementing and informing parents about the NN intervention, staff described parents of young children as navigating a hectic period, perhaps preferring faster or more immediately accessible formats of health information (eg, apps) rather than exploring a resource [34]. Another factor may be the wide availability of other digital platforms in recent years. In the qualitative evaluation from our research group, staff reported that parents now access dietary information from multiple sources, including government-supported websites, commercial apps, and social media platforms [34]. The competing availability of these alternative platforms may reduce engagement with NN’s website format, even though it was positively rated.

Similar to the results of this study, Borghouts et al [35] identified time limitations, forgetfulness, and difficulties in integrating digital health tools into daily routines as barriers to engagement in their review of digital mental health interventions. They noted that participants in self-guided interventions often struggle with engagement, suggesting that structured user guidance could be beneficial [35]. Given these challenges, self-guided e-learning resources like NN might benefit from more structured use instructions. Clearer, organized guidance might enhance user engagement, helping users better integrate the e-learning resource into their daily lives.

Moreover, reviews on engagement strategies for digital mental health interventions have suggested features such as gamification and prompts as measures that may help promote engagement [36,37]. Gamification may include features such as progress bars, streak counters, or achievement badges [38]. For the NN e-learning intervention, gamification features could include tracking streaks showing the number of times or days the user has visited the resource or progress bars showing the number of thematic films completed and perhaps influence interest and motivation to engage with the resource. In addition, participants in this study received email newsletters containing content from the e-learning intervention to encourage engagement. In a mixed methods study evaluating engagement with a digital health intervention focused on type 2 diabetes management, Alkhaldi et al [39] reported that email prompts were significantly associated with visits to the digital health intervention, whereas text messaging was not. Participants in their study preferred short and clear prompts, with one noting that clear descriptions of the linked content would be a useful feature [39]. In the NN intervention, the email newsletters were designed to be brief and clear. However, including information on the estimated time required to view a video or read a text might help parents consider using the resource despite time constraints.

Nevertheless, engaging participants in digital health interventions remains challenging. A narrative review proposed human support as a program feature that may influence engagement with digital mental health interventions [36]. However, the optimal type of support and participants most likely to benefit remain unclear. In real-world settings, a multipronged approach may be effective. Within the NN intervention, human support may be a feasible and appropriate strategy, as the e-learning resource is delivered through MCHs and ECECs. Ensuring that these professionals are familiar with the intervention and equipped to deliver nutritional guidance and support may enhance the perceived relevance and usefulness of the intervention among parental users. However, further efforts are needed to understand facilitators and barriers of use and engagement with digital food and nutrition interventions. Qualitative methods could provide deeper insights into parents’ motivations for engaging with such interventions [40], as well as reasons for nonparticipation [41]. It could also provide greater insight into features needed to promote the use, acceptability, feasibility, and appropriateness of digital nutrition resources. Another strategy could have been to involve parents in the development process. Co-design approaches involve engaging end users and key stakeholders early in intervention development to embed their needs and values into the intervention within the design while identifying and addressing potential barriers for adoption [42]. Although parents and health care nurses were involved during development for some of the original interventions underpinning NN, a more explicit cocreation design process could have included parents with diverse socioeconomic and cultural backgrounds to inform decisions about content and modes of delivery in the NN intervention, with the aim of improving fit across the full target population. Such an approach could have helped ensure that the intervention was fully aligned with what parents find realistic and useful in everyday life.

### Strengths and Limitations

A key strength of this study is its real-world setting, where participants had free access to the NN e-learning resource and could engage with it at their discretion. This approach provides a more authentic reflection of how such resources are accessed and used in everyday contexts compared to controlled trial settings.

This study also has some limitations. The proportion of participants who responded to the follow-up questionnaire was low (48/179, 27%). Those who did respond had a higher educational level than those lost to follow-up, raising concern about self-selection bias, potentially making the results more reflective of individuals with higher educational attainment. We originally intended to examine differences in implementation and use outcomes by sociodemographic characteristics, but the small, homogeneous sample made this analysis unfeasible. Additionally, it is important to acknowledge that a large proportion of participants did not complete the follow-up questionnaire, resulting in limited insights into their experiences with the resource and uncertainty regarding overall use and acceptance. The high loss to follow-up should also be interpreted, considering the study design. The use of a hybrid implementation-effectiveness design allowed the intervention to be accessed under real-world conditions, in which participation was voluntary, and follow-up was digital through email prompts. Nevertheless, relying solely on digital modalities for follow-up may have contributed to lower retention and completion of follow-up surveys. Alternative designs, such as feasibility studies, could have helped identify and address retention challenges before moving forward with implementation.

Another limitation that may have influenced findings on resource use is that participants in NN were recruited at any time between early pregnancy and child age 2 years. Although participants had 7 months of access to the resource at the time of the survey, the number and type of newsletters they received during that period differed according to the status of pregnancy or parent at enrollment. As the newsletters contained links to the resource, this variation may have influenced how often participants were prompted to visit the resource. Furthermore, the original AIM, IAM, and FIM measures were modified for this study, including removal and rewording of items, which limits content validity and direct comparison with other studies using the fully validated instruments. Although validated measures exist for related constructs such as usability, user experiences, or technology acceptance, there are few brief, validated instruments that assess acceptability, appropriateness, and feasibility as defined within the implementation outcomes framework by Proctor et al [17]. Nevertheless, using alternative validated measures such as the user version of the Mobile Application Rating Scale [43], or the System Usability Scale [44], could have provided more valuable insights into aspects of intervention engagement, functionality, or perceived usability, particularly to inform refinements of the NN intervention prior to potential scale-up.

Future research should explore different approaches to assess these constructs among users of digital health interventions.

### Conclusions

The NN e-learning resource was rated positively among those who responded to the survey, on items assessing perceived resource liking, appreciation, appropriateness, and whether it seemed doable and easy to use. Among respondents, access and use of the resource were limited, highlighting challenges of engaging expectant parents and parents of infants and toddlers with self-guided interventions in real-world settings.

Future research should examine the effectiveness of digital interventions in improving healthy eating behaviors during the first 1000 days, assess strategies needed to engage users with such interventions, and refine approaches to assessing early-phase implementation outcomes, including the measurements of acceptability, feasibility, and appropriateness, and their potential overlap.

## Supplementary material

10.2196/84277 Multimedia Appendix 1 Screenshots from the Nutrition Now e-learning resource.

10.2196/84277 Multimedia Appendix 2 Survey questions included in this study.

10.2196/84277 Multimedia Appendix 3 Internal consistency, correlations, and median participant ratings for adapted items from the Acceptability of Intervention Measure, Feasibility of Intervention Measure, and Intervention Appropriateness Measure in the Nutrition Now study.

10.2196/84277 Checklist 1 CHERRIES checklist.

## Abbreviations

AIM: Acceptability of Intervention Measure
CHERRIES: Checklist for Reporting Results of Internet E-Surveys
ECEC: Early Childhood Education and Care
FIM: Feasibility of Intervention Measure
IAM: Intervention Appropriateness Measure
MCH: Maternal and Child Health
mHealth: mobile health
NN: Nutrition Now
