# Supplementary material for: A Formative Evaluation of Parental Perceptions Related to Acceptability, Appropriateness, Feasibility, and Reported Use of an e-Learning Resource Targeting Diet in the First 1000 Days: Survey Study
Source: JMIR Form Res. 2026 Apr 28;10:e84277. doi: 10.2196/84277 (PMC13123635; doi:10.2196/84277)
Supplement: Multimedia Appendix 1 [file formative-v10-e84277-s001.pptx]

## Slide 1
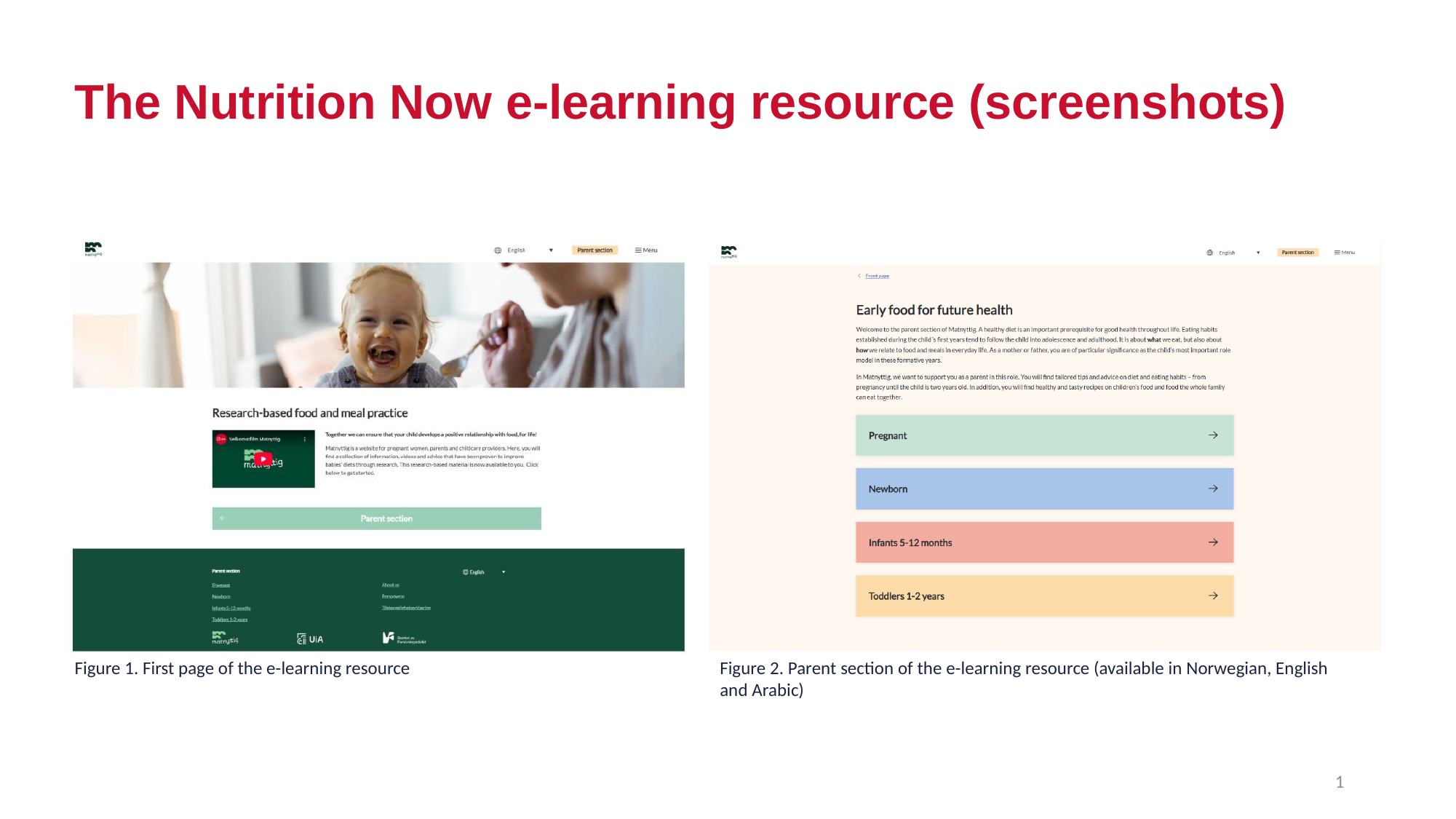

# The Nutrition Now e-learning resource (screenshots)
Figure 2. Parent section of the e-learning resource (available in Norwegian, English and Arabic)
Figure 1. First page of the e-learning resource
1

## Slide 2
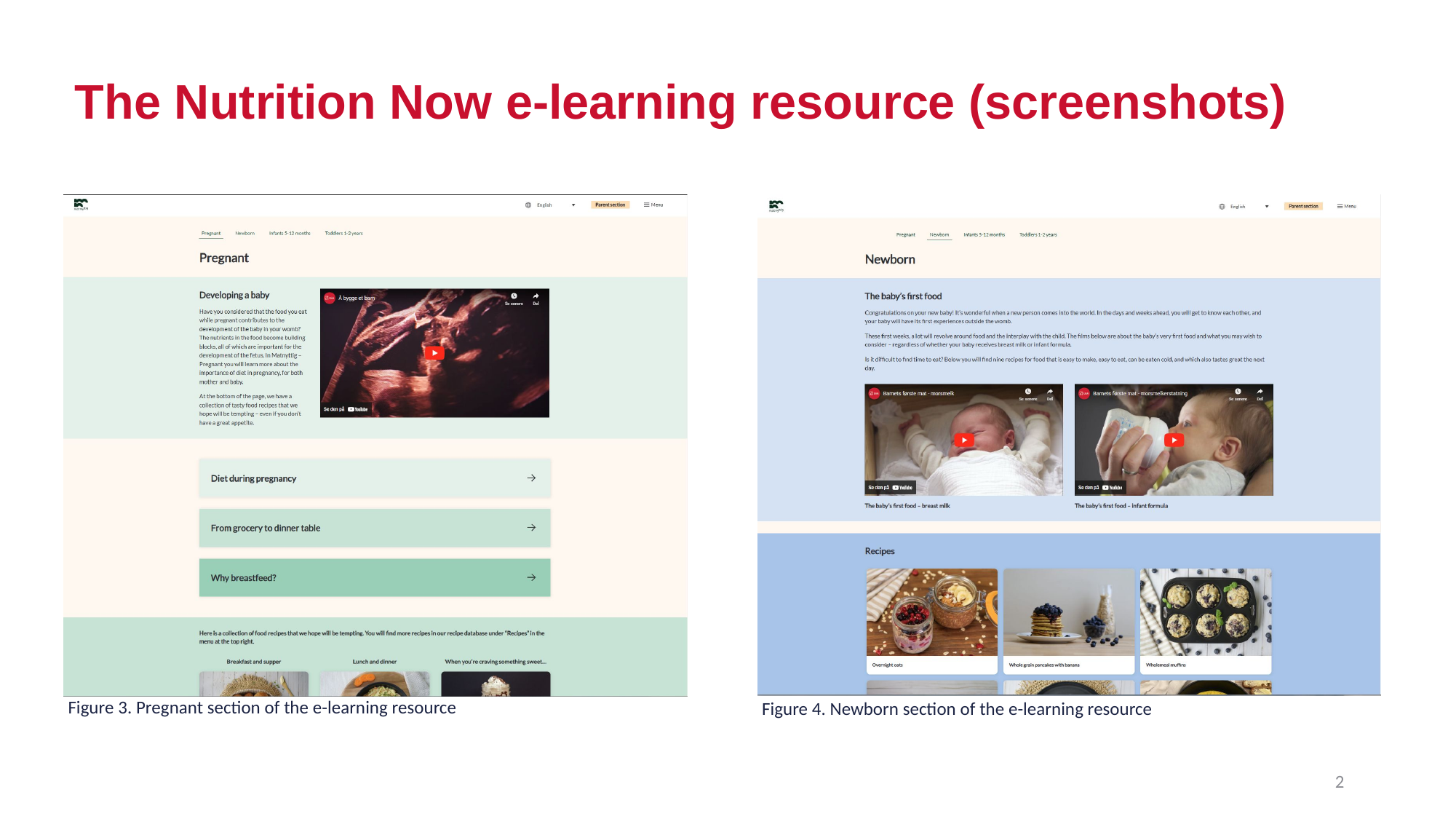

# The Nutrition Now e-learning resource (screenshots)
Figure 3. Pregnant section of the e-learning resource
Figure 4. Newborn section of the e-learning resource
2

## Slide 3
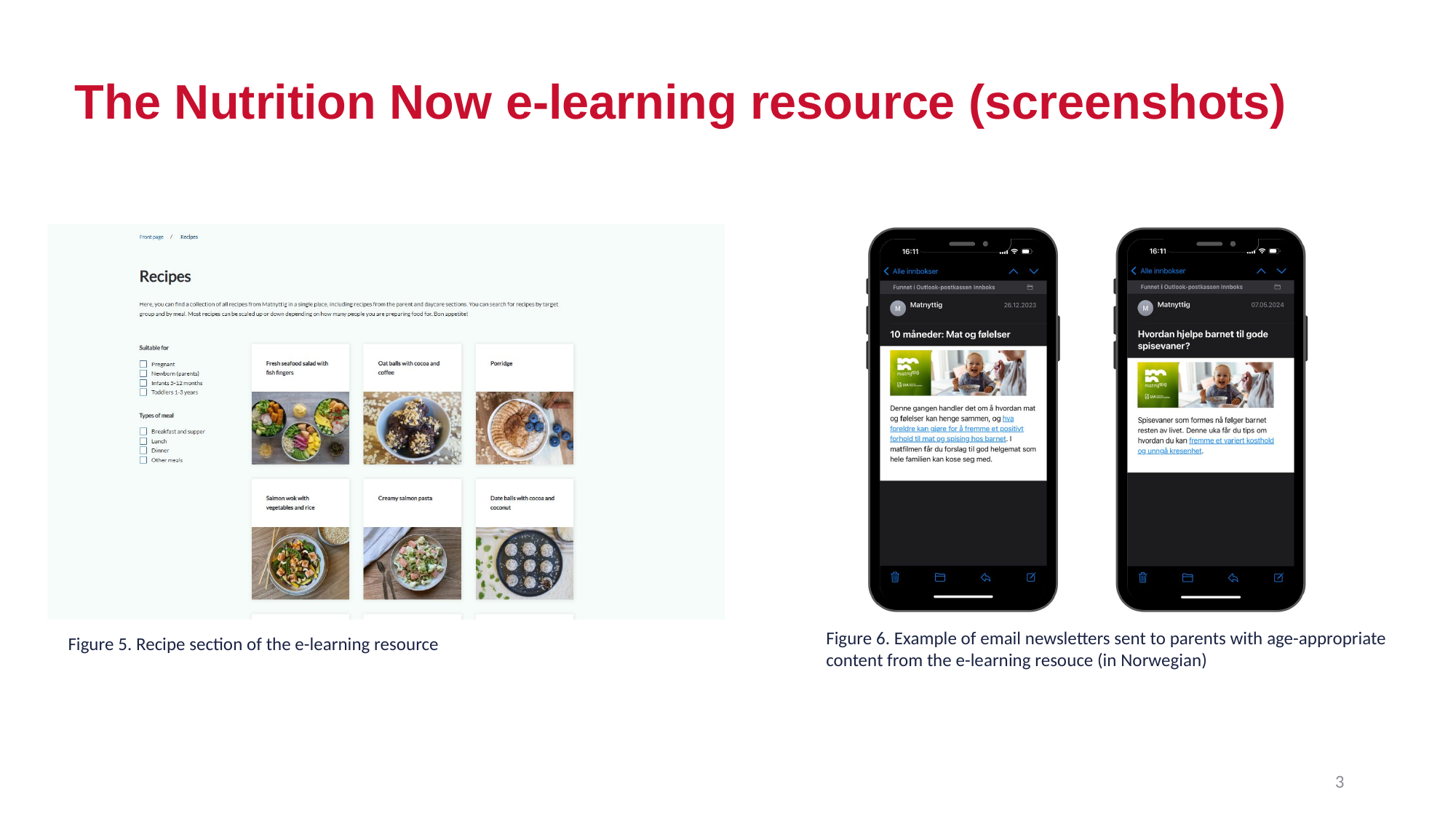

# The Nutrition Now e-learning resource (screenshots)
Figure 6. Example of email newsletters sent to parents with age-appropriate content from the e-learning resouce (in Norwegian)
Figure 5. Recipe section of the e-learning resource
3
