# Supplementary material for: A Formative Evaluation of Parental Perceptions Related to Acceptability, Appropriateness, Feasibility, and Reported Use of an e-Learning Resource Targeting Diet in the First 1000 Days: Survey Study
Source: JMIR Form Res. 2026 Apr 28;10:e84277. doi: 10.2196/84277 (PMC13123635; doi:10.2196/84277)
Supplement: Multimedia Appendix 2 [file formative-v10-e84277-s002.docx]

**Table S1**. Survey questions from the web-based background questionnaire administered at baseline.

| Q1. | What gender are you?   1. Male 2. Female 3. Other    1. Specify, if you wish |
| --- | --- |
| Q2. | What is your current marital status?   1. Single 2. In a relationship (not cohabitant/not married) 3. Cohabitant 4. Married 5. Divorced/separated 6. Widow/widower 7. Other    1. Specify, if you wish |
| Q3. | What is currently your main activity?   1. Full-time work 2. Part-time work 3. Staying at home 4. Sick leave 5. Leave of absence 6. Laid off 7. Disabled 8. In rehabilitation 9. Student/school pupil 10. Unemployed 11. Other     1. Specify, if you wish |
| Q4. | What type of education do you have? Select your highest level of education.   1. Less than 9–10 years of primary school 2. Primary and lower secondary school 3. Upper secondary school 4. Upper secondary school – vocational subject 5. University/university college up to 4 years 6. University/university college more than 4 years 7. Other education    1. Specify, if you wish |
| Q5. | What level of education does the other parent have?   1. Less than 9–10 years of primary school 2. Primary and lower secondary school 3. Upper secondary school 4. Upper secondary school – vocational subject 5. University/university college up to 4 years 6. University/university college more than 4 years 7. Other education    1. Specify. If you wish 8. Unsure/Don't know |
| Q6. | In which country were you born?   1. Response options consisted of a 201-category list consisting of different countries. The last response option was “Other, specify”. |
| Q7. | How many people live in your household? (Include yourself).   1. Open response option. |
| Q8. | What was your household's total gross annual income (before tax) over the past year? (Include child support, unemployment benefit, cash benefits, etc.)   1. Under NOK 150,000 2. NOK 150,000–199,999 3. NOK 200,000–299,999 4. NOK 300,000–399,999 5. NOK 400,000–499,999 6. NOK 500,000–599,999 7. NOK 600,000–699,999 8. NOK 700,000–799,999 9. NOK 800,000–899,999 10. NOK 900,000–999,999 11. NOK 1,000,000–1,099,999 12. NOK 1,100,000–1,199,999 13. NOK 1,200,000–1,299,999 14. NOK 1,300,000–1,399,999 15. 1,400,000 or above 16. Do not wish to answer |
| Q9. | I live in:   1. An urban area (city/town/suburbs with at least 200 people and a short distance between houses) 2. A rural area (outside of urban areas) |

**Table S2.** Survey questions from the web-based questionnaire administered 7 months post-enrolment, evaluating implementation outcomes and use.

| Q1. | Have you visited the Nutrition Now online resource?   1. Yes, quite a lot 2. Yes, a bit 3. No |
| --- | --- |
| Q2. | Why did you not visit the Nutrition Now online resource?   1. Didn't have time 2. Had technical problems 3. Lost interest 4. Other    1. If other, describe |
| Q3. | The next questions are about your experiences with Nutrition Now.  To what extent do you agree with the following statements: |
| (AIM, Item 3) | I like the Nutrition Now resource |
| (AIM, Item 4) | I appreciate Nutrition Now as a new resource |
| (IAM, Item 1) | Nutrition Now seems appropriate as a source of information regarding meals for my family |
| (FIM, Item 3) | The Nutrition Now resource seems doable |
| (FIM, Item 4) | The Nutrition Now resource seems easy to use |
|  | 1. Completely disagree 2. Disagree 3. Neither agree nor disagree 4. Agree 5. Completely agree |
| Q4. | Have you watched the films in the Nutrition Now online resource?   - The theme films - Food films/Recipe films  1. Yes 2. No 3. Don't know |
| Q5. | Have you made any of the food recipes from the Nutrition Now online resource?   1. Yes 2. No 3. Don't know |

Abbreviations: AIM, Acceptability of Intervention Measure; IAM, Appropriateness of Intervention Measure; FIM, Feasibility of Intervention Measure
