# Supplementary material for: A Formative Evaluation of Parental Perceptions Related to Acceptability, Appropriateness, Feasibility, and Reported Use of an e-Learning Resource Targeting Diet in the First 1000 Days: Survey Study
Source: JMIR Form Res. 2026 Apr 28;10:e84277. doi: 10.2196/84277 (PMC13123635; doi:10.2196/84277)
Supplement: Multimedia Appendix 3 [file formative-v10-e84277-s003.docx]

**Table S1.** Internal consistency and Spearman correlations for adapted items from the AIM, FIM, and IAM measures.

| Measure | Items (n) | AIM | FIM | IAM | Cronbach’s α |
| --- | --- | --- | --- | --- | --- |
| AIM (acceptability) | 2 | - |  |  | 0.891 |
| FIM (feasibility) | 2 | 0.535* | - |  | 0.515 |
| IAM (appropriateness) | 1 | 0.569* | 0.376** | - | - |

Abbreviations: AIM=Acceptability of Intervention Measure, FIM=Feasibility of Intervention Measure, IAM=Intervention Appropriateness Measure
*p-value < .001
**p-value=.008
Acceptability was assesses using adapted items 3 and 4 from the AIM scale (“I like the Nutrition Now resource” and “I appreciate the Nutrition Now as a new resource”), feasibility using adapted items 3 and 4 from the FIM scale (“The Nutrition Now resource seems doable” and “The Nutrition Now resource seems easy to use”), and appropriateness using adapted item 1 from the IAM scale (“Nutrition Now seems appropriate as a source of information regarding meals for my family”). Cronbach’s alpha is reported for the two-item AIM and FIM measures. Spearman rho correlations were calculated between the IAM item and the mean scores of the AIM and FIM items.

**Table S2**. Median ratings for adapted items based on the Acceptability of Intervention Measure (AIM), Feasibility of Intervention Measure (FIM) and Intervention Appropriateness Measure (IAM) among participants in Nutrition Now (n=48).

|  |  | Median (25^th:^75^th^) | Min-Max |
| --- | --- | --- | --- |
| **AIM** | Item 3: I like the Nutrition Now resource | 4 (3:4) | 1-5 |
|  | Item 4: I appreciate Nutrition Now as a new resource | 4 (3:4) | 1-5 |
| **FIM** | Item 3: The Nutrition Now resource seems doable | 4 (3:4) | 3-5 |
|  | Item 4: The Nutrition Now resource seems easy to use | 4 (3:4) | 2-5 |
| **IAM** | Item 1: Nutrition Now seems appropriate as a source of information regarding meals for my family | 4 (3:4) | 1-5 |
