# Supplementary material for: A Formative Evaluation of Parental Perceptions Related to Acceptability, Appropriateness, Feasibility, and Reported Use of an e-Learning Resource Targeting Diet in the First 1000 Days: Survey Study
Source: JMIR Form Res. 2026 Apr 28;10:e84277. doi: 10.2196/84277 (PMC13123635; doi:10.2196/84277)
Supplement: Checklist 1 [file formative-v10-e84277-s004.docx]

**Checklist for Reporting Results of Internet E-Surveys (CHERRIES)**

| ***Checklist Item*** | ***Explanation*** |
| --- | --- |
| Describe survey design | *Describe target population, sample frame. Is the sample a convenience sample? (In “open” surveys this is most likely.)*  Descriptive study using a web-based survey to explore parental perceptions related to the acceptability, appropriateness, feasibility and reported use of a digital e-learning resource targeting diet in the first 1000 days. The target population comprised expecting parents and parents of children aged 0-2 years who had received access to the Nutrition Now e-learning resource and were invited to complete a web-based follow-up survey seven months after receiving access. |
| IRB approval | *Mention whether the study has been approved by an IRB*  This study is part of the Nutrition Now project, approved by the regional committees for Medical Research Ethics South East Norway (REC, reference number: 32248), the University of Agder’s Faculty Ethical Committee (FEC), and the Norwegian Data Protection Service (NSD, reference number: 847590). |
| Informed consent | *Describe the informed consent process. Where were the participants told the length of time of the survey, which data were stored and where and for how long, who the investigator was, and the purpose of the study?* All participants who wished to take part in the Nutrition Now study were directed to the study’s registration website where participants were informed about the study’s aim and their participation rights, including their right to withdraw from the study at any time. Electronic informed consent was obtained when participants chose to sign up for the study. All data was stored on secure servers. |
| Data protection | *If any personal information was collected or stored, describe what mechanisms were used to protect unauthorized access*  All data was stored on a secure, password-protected served (TSD). Data were pseudonymized using numerical identifiers, and access to identifiable information was restricted to members of the research team. |
| Development and testing | *State how the survey was developed, including whether the usability and technical functionality of the electronic questionnaire had been tested before fielding the questionnaire.*  The survey was developed using the web-based survey tool Nettskjema. The survey was piloted on desktop and mobile devices by the authors involved in development of questionnaires (NGO, FNV, ERH, NCØ, ACM, and CH). |
| Open survey versus closed survey | *An “open survey” is a survey open for each visitor of a site, while a closed survey is only open to a sample which the investigator knows (password-protected survey).* Closed survey |
| Contact mode | *Indicate whether or not the initial contact with the potential participants was made on the Internet. (Investigators may also send out questionnaires by mail and allow for Web-based data entry.)*  Initial contact with participants occurred both offline and online as participants were recruited to the intervention through maternal and child healthcare centers, early childhood education and care centers, municipal websites, and social media. Participants who had received access to the e-learning resource were invited to complete the follow-up survey via email, which included a link to the web-based questionnaire. |
| Advertising the survey | *How/where was the survey announced or advertised? Some examples are offline media (newspapers), or online (mailing lists – If yes, which ones?) or banner ads (Where were these banner ads posted and what did they look like?). It is important to know the wording of the announcement as it will heavily influence who chooses to participate. Ideally the survey announcement should be published as an appendix.*  The survey was distributed through e-mail to participants in the Nutrition Now study living in the intervention municipality who had registered to the study and gained access to the e-learning resource. |
| Web/E-mail | *State the type of e-survey (eg, one posted on a Web site, or one sent out through e-mail). If it is an e-mail survey, were the responses entered manually into a database, or was there an automatic method for capturing responses?*  Surveys were distributed via email, which directed participants to the survey platform. Responses were collected automatically within the Nettskjema platform. |
| Context | *Describe the Web site (for mailing list/newsgroup) in which the survey was posted. What is the Web site about, who is visiting it, what are visitors normally looking for? Discuss to what degree the content of the Web site could pre-select the sample or influence the results. For example, a survey about vaccination on a anti-immunization Web site will have different results from a Web survey conducted on a government Web site*  The survey was distributed to participants who had access to the e-learning resource, which focus on diet during the first 1000 days. Participants answering the follow-up survey may represent parents with an interest in pregnancy and child nutrition. |
| Mandatory/voluntary | *Was it a mandatory survey to be filled in by every visitor who wanted to enter the Web site, or was it a voluntary survey?*  The follow-up survey was voluntary to complete. |
| Incentives | *Were any incentives offered (eg, monetary, prizes, or non-monetary incentives such as an offer to provide the survey results)?*  No incentives were offered. |
| Time/Date | *In what timeframe were the data collected?*  Participants were recruited to the study between October 2022 and May 2023 and would receive the follow-up survey seven months after enrolling to the study. |
| Randomization of items or questionnaires | *To prevent biases items can be randomized or alternated*  Questions were not randomized. |
| Adaptive questioning | *Use adaptive questioning (certain items, or only conditionally displayed based on responses to other items) to reduce number and complexity of the questions.*  Adaptive questioning was applied for two of the questions in the survey, see Additional file 2. |
| Number of Items | *What was the number of questionnaire items per page? The number of items is an important factor for the completion rate.*  Number of questions per page were 1-3 depending on adaptive questioning. In total, depending on adaptive questioning, participants were asked 4-6 questions. |
| Number of screens (pages) | *Over how many pages was the questionnaire distributed? The number of items is an important factor for the completion rate.*  4 |
| Completeness check | *It is technically possible to do consistency or completeness checks before the questionnaire is submitted. Was this done, and if “yes”, how (usually JAVAScript)? An alternative is to check for completeness after the questionnaire has been submitted (and highlight mandatory items). If this has been done, it should be reported. All items should provide a non-response option such as “not applicable” or “rather not say”, and selection of one response option should be enforced.* All questionnaire items were mandatory, and participants were required to complete each item before submitting the questionnaire. |
| Review step | *State whether respondents were able to review and change their answers (eg, through a Back button or a Review step which displays a summary of the responses and asks the respondents if they are correct).* Respondents could review and change their answers by navigating back through the survey. |
| Unique site visitor | *If you provide view rates or participation rates, you need to define how you determined a unique visitor. There are different techniques available, based on IP addresses or cookies or both*  Unique respondents were identified using individual numerical identifiers assigned at enrollment. These identifiers were used to link responses to follow-up surveys across timepoints. |
| View rate (Ratio of unique survey visitors/unique site visitors) | *Requires counting unique visitors to the first page of the survey, divided by the number of unique site visitors (not page views!). It is not unusual to have view rates of less than 0.1 % if the survey is voluntary.*  Not applicable. |
| Participation rate (Ratio of unique visitors who agreed to participate/unique first survey page visitors) | *Count the unique number of people who filled in the first survey page (or agreed to participate, for example by checking a checkbox), divided by visitors who visit the first page of the survey (or the informed consents page, if present). This can also be called “recruitment” rate.*  Not applicable. |
| Completion rate (Ratio of users who finished the survey/users who agreed to participate) | *The number of people submitting the last questionnaire page, divided by the number of people who agreed to participate (or submitted the first survey page). This is only relevant if there is a separate “informed consent” page or if the survey goes over several pages. This is a measure for attrition. Note that “completion” can involve leaving questionnaire items blank. This is not a measure for how completely questionnaires were filled in. (If you need a measure for this, use the word “completeness rate”.)* 48 out of 179 eligible participants (27%) completed the web-based follow-up questionnaire administered 7 months after enrolment. |
| Cookies used | *Indicate whether cookies were used to assign a unique user identifier to each client computer. If so, mention the page on which the cookie was set and read, and how long the cookie was valid. Were duplicate entries avoided by preventing users access to the survey twice; or were duplicate database entries having the same user ID eliminated before analysis? In the latter case, which entries were kept for analysis (eg, the first entry or the most recent)?* Duplicate entries having the same user ID were eliminated before analysis. The first entry was kept for analysis. |
| IP check | *Indicate whether the IP address of the client computer was used to identify potential duplicate entries from the same user. If so, mention the period of time for which no two entries from the same IP address were allowed (eg, 24 hours). Were duplicate entries avoided by preventing users with the same IP address access to the survey twice; or were duplicate database entries having the same IP address within a given period of time eliminated before analysis? If the latter, which entries were kept for analysis (eg, the first entry or the most recent)?* No additional exclusions were performed based in IP address. |
| Log file analysis | *Indicate whether other techniques to analyze the log file for identification of multiple entries were used. If so, please describe.*  No other techniques were used. |
| Registration | *In “closed” (non-open) surveys, users need to login first and it is easier to prevent duplicate entries from the same user. Describe how this was done. For example, was the survey never displayed a second time once the user had filled it in, or was the username stored together with the survey results and later eliminated? If the latter, which entries were kept for analysis (eg, the first entry or the most recent)?*  The user name was stored together with the survey results and a unique identifier for each response. The username was eliminated before analyses, and if there were duplicate entries, the first entry was kept. |
| Handling of incomplete questionnaires | *Were only completed questionnaires analyzed? Were questionnaires which terminated early (where, for example, users did not go through all questionnaire pages) also analyzed?*  Only completed questionnaires were analyzed, as participants had to complete the survey to submit responses. |
| Questionnaires submitted with an atypical timestamp | *Some investigators may measure the time people needed to fill in a questionnaire and exclude questionnaires that were submitted too soon. Specify the timeframe that was used as a cut-off point, and describe how this point was determined.*  No cut-off time was used in screening responses. |
| Statistical correction | *Indicate whether any methods such as weighting of items or propensity scores have been used to adjust for the non-representative sample; if so, please describe the methods.*  No statistical correction was used for the analyses |

This checklist has been modified from Eysenbach G. Improving the quality of Web surveys: the Checklist for Reporting Results of Internet E-Surveys (CHERRIES). J Med Internet Res. 2004 Sep 29;6(3):e34 [erratum in J Med Internet Res. 2012; 14(1): e8.]. Article available at [https://www.jmir.org/2004/3/e34](https://www.jmir.org/2004/3/e34/)/; erratum available <https://www.jmir.org/2012/1/e8/>. Copyright ©Gunther Eysenbach. Originally published in the [Journal of Medical Internet](http://www.jmir.org) Research, 29.9.2004 and 04.01.2012.

This is an open-access article distributed under the terms of the Creative Commons Attribution License (<https://creativecommons.org/licenses/by/2.0/>), which permits unrestricted use, distribution, and reproduction in any medium, provided the original work, first published in the Journal of Medical Internet Research, is properly cited.
